# Identification and Biosynthesis of Tropodithietic Acid by *Janthinobacterium* sp.

**DOI:** 10.3390/ijms27094052

**Published:** 2026-04-30

**Authors:** Sergei I. Belikov, Yuliya Panova, Alina Belikova, Lubov Chernogor

**Affiliations:** 1 Limnological Institute, Siberian Branch of the Russian Academy of Sciences, Ulan-Batorskaya Str., 3, Irkutsk 664033, Russia; sergeibelikov47@gmail.com (S.I.B.);; 2 Siberian Federal University, Svobodny Prosp., 79, Krasnoyarsk 660041, Russia

**Keywords:** *Janthinobacterium* sp. PLB04, tropodithietic acid, secondary metabolites, biosynthetic gene cluster, tda operon, antibiotic production, comparative genomics, Lake Baikal

## Abstract

Tropodithietic acid (TDA) is a sulfur-containing secondary metabolite with pronounced antimicrobial activity that has been primarily described in marine Alphaproteobacteria of the *Roseobacter* clade. Despite extensive studies of these bacteria, the occurrence and genetic organization of the TDA biosynthetic pathway in other bacterial groups remain poorly understood. In this study, we report the production of TDA by the freshwater bacterium *Janthinobacterium* sp. PLB04 isolated from diseased cell cultures of the primmorphs from the Baikal sponge *Lubomirskia baikalensis*. The presence of a TDA biosynthetic gene cluster homologous to the canonical tda operon previously described in the marine *Roseobacter* clade was found in *Janthinobacterium* sp. PLB04 by genome mining with bioinformatic analysis. However, comparative analysis of the cluster architecture demonstrated the absence of the gene tdaC in the *Janthinobacterium* sp. PLB04 genome. Despite this difference, the strain retained the ability to synthesize TDA. TDA was extracted from the culture medium and identified using chromatographic and MALDI-TOF mass spectrometric analysis. These results suggest that tdaC may not be strictly required for TDA biosynthesis in this strain and may be functionally replaced with alternative enzymatic steps or functional redundancy within the pathway. The discovery of TDA production in a freshwater *Janthinobacterium* strain expands the known phylogenetic and ecological diversity of TDA-producing bacteria and provides new insights into the plasticity of the TDA biosynthetic gene cluster.

## 1. Introduction

In recent years, there has been an exponential increase in the resistance of pathogens to most known classes of modern antibiotics. This trend drives the urgent search for new antimicrobial compounds effective against key clinical pathogens, as well as the identification of novel producers of promising antibiotics. Microorganisms, particularly bacteria, represent one of the most important sources of biologically active secondary metabolites, including many clinically used antibiotics. Unique and understudied ecosystems, such as microbial communities associated with freshwater sponges, may serve as promising reservoirs of previously unknown producers of bioactive compounds.

Tropodithietic acid, a known antibiotic, is a sulfur-containing compound with a unique structure consisting of a tropone-2-carboxylic acid moiety linked to a dithiete group. TDA is known as a secondary metabolite produced primarily by marine bacteria. Numerous studies have demonstrated that TDA exhibits strong inhibitory activity against a wide range of bacteria, including taxa pathogenic to humans and marine organisms. This activity has been demonstrated against both Gram-positive and Gram-negative bacteria [1,2]. In particular, activity against marine pathogens such as Vibrio species has been reported [3]. TDA is thought to exert its antimicrobial effect, at least in part, through disruption of the proton motive force [4]. It was first isolated and chemically characterized from *Roseobacter* sp. [5]. To date, confirmed TDA producers have been reported predominantly among marine Alphaproteobacteria of the *Roseobacter* group, including species of the genera *Phaeobacter*, *Ruegeria*, and *Pseudovibrio*, was isolated from marine sponges [1,3,6]. These bacteria are typically associated with marine environments such as algal surfaces, invertebrates, or aquaculture systems [7]. In contrast, reports of TDA production outside this phylogenetic group remain extremely limited.

The production of TDA is regulated by quorum sensing mechanisms [8]. In addition, TDA has been proposed to act as a signaling molecule in microbial communities [9]. Genetic analyses revealed that TDA production is associated with a specific biosynthetic gene cluster, commonly referred to as the tda operon, which typically includes genes tdaA–F [10]. Disruption of these genes has been shown to abolish TDA production and antimicrobial activity [10]. In addition, the TDA biosynthetic pathway is closely linked to phenylacetate catabolism suggesting that the antibiotic is synthesized via modifications of intermediates from aromatic compound metabolism [11].

In this study, we report for the first time the production of TDA by the strain *Janthinobacterium* sp. PLB04 isolated from diseased cell cultures of the primmorphs from the Baikal freshwater sponge *L. baikalensis*. The aim of the study was to conduct a comparative analysis of TDA operons with the structure of the biosynthetic gene cluster in the *Janthinobacterium* sp. PLB04 strain in comparison with *Roseobacter* clade. The results of this study will further expand our understanding of TDA biosynthetic pathways and the genetic organization of the biosynthetic pathway in other types of bacteria.

## 2. Results

### 2.1. Isolation of the Strain Janthinobacterium sp. PLB04

**Figure 1:**
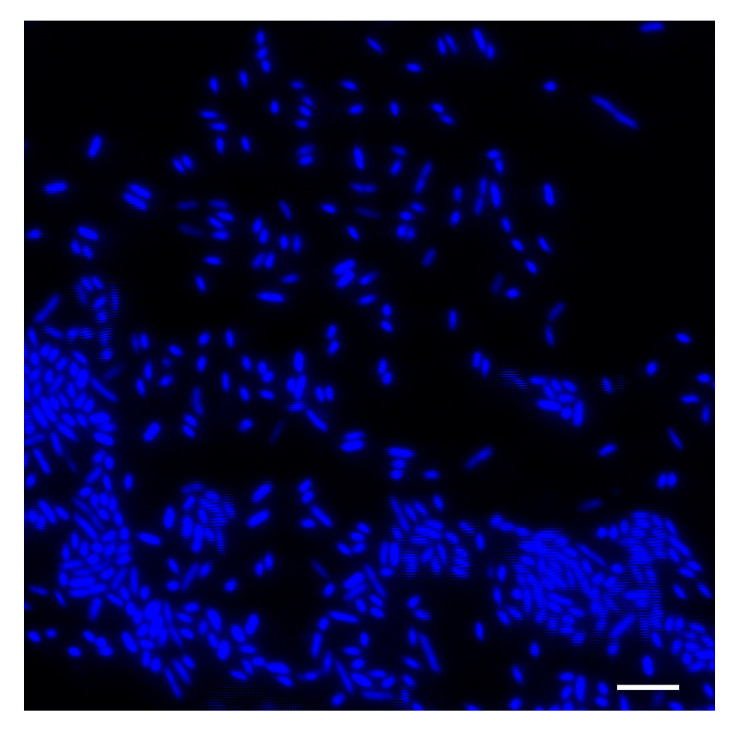
Fluorescence microscopy of the strain *Janthinobacterium* sp. PLB04. Bacterial cells were stained with NucBlue Live ReadyProbes reagent for fluorescence microscopy. Bacteria are shown with a blue color. Scale bar: 10 µm.

The bacteria were Gram-negative, psychrotolerant, aerobic and motile. The bacteria were rod-shaped with rounded ends, and had a length to 2.0 µm and a width from 0.3 µm to 1.0 µm, with twitching motility and flagella (Figure 1).

Bacterial cells grew and produced a purple pigment on the 2nd–3rd day of cultivation at temperature 21 °C. Under static (non-shaking) conditions, a dense violet biofilm formed at the air–liquid inter-face due to violacein production. During long-term (more than 30 days) cultivation, the culture medium gradually acquired a brown coloration, which has been reported as an indicator of tropodithietic acid (TDA) production in *Roseobacter* species [10].

### 2.2. Identification of TDA Biosynthetic Gene Cluster in the Genome of Janthinobacterium sp. PLB04

**Figure 2:**
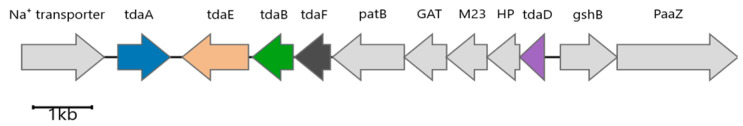
Organization of the TDA-related gene cluster in the *Janthinobacterium* sp. PLB04. The locus includes homologs of tdaA, tdaB, tdaD, tdaE, and tdaF. The genes tdaA, tdaB, tdaE, and tdaF are arranged in a contiguous block, while tdaD is located separately. No tdaC homolog was identified. Additional genes within the region include patB, GAT, M23 peptidase, hypothetical protein, a gshB-like gene, and paaZ. A Na^+^ transporter gene is located upstream.

Genomic analysis of *Janthinobacterium* sp. PLB04 using antiSMASH revealed a potential biosynthetic region with low similarity to known TDA clusters, prompting a detailed investigation of the genomic basis of its biosynthesis. Targeted BLAST (version 2.17.0) searches with reference TdaA–F protein sequences confirmed the presence of homologs of tdaA, tdaB, tdaD, tdaE, and tdaF within this region, while no tdaC homolog was detected (Figure 2). Taken together with the experimentally confirmed production of TDA (see below), these findings support the interpretation that this region represents a TDA-related biosynthetic locus.

In addition to the core genes, the locus contains neighboring genes, including patB, a glutamine amidotransferase, a gshB-like gene, and paaZ2. Despite extensive homology searches, using both BLASTp and tBLASTn approaches, no convincing tdaC homolog was identified in the *Janthinobacterium* sp. PLB04 genome. We found no unannotated open reading frames or intergenic regions within the identified locus could plausibly correspond to a highly divergent tdaC-like gene. These data support the conclusion that this gene is truly missing and not missed due to annotation limitations.

Homologous TDA-related loci were identified in the *Janthinobacterium* sp. PLB04, *J. lividum* EIF2, *J. violaceum* LB2P70, and *J. amylolyticum* RT4P48. All analyzed strains exhibited the same gene organization, comprising a contiguous tdaA–tdaB–tdaE–tdaF block and a spatially separated tdaD gene, with no detectable tdaC homolog. No additional TDA-related loci were found in the analyzed genomes.

### 2.3. Comparative Analysis of TDA Gene Cluster Organization

**Figure 3:**
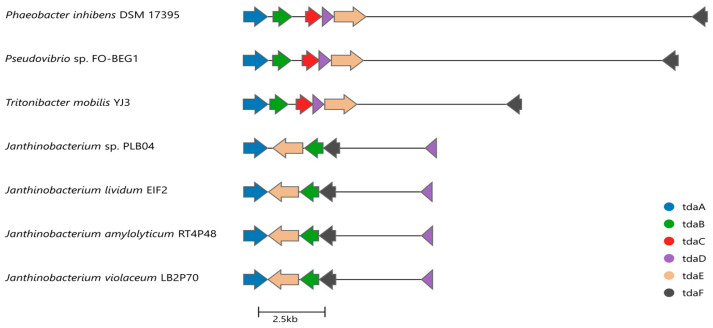
Comparative organization of TDA biosynthetic gene clusters in *Janthinobacterium* and representative alphaproteobacterial strains. Schematic representation of TDA-related gene loci in the *Janthinobacterium* and Alphaproteobacteria. Arrows indicate coding sequences and their orientation; colors denote homologous genes.

We found that, in representative Alphaproteobacteria (*P. inhibens* DSM 17395, *Pseudovibrio* sp. FO-BEG1, *Tritonibacter mobilis* YJ3), the core genes tdaA–tdaE are co-localized, whereas tdaF is located outside the main gene block (Figure 3). In the strains *Janthinobacterium*, the organization consists of a contiguous tdaA–tdaB–tdaE–tdaF block and a spatially separated tdaD gene (Figure 3).

In the alphaproteobacterial strains shown and the core biosynthetic genes tdaA–tdaE are co-localized, while tdaF is located separately. In *Janthinobacterium* strains, tdaA, tdaB, tdaE, and tdaF form a conserved block, whereas tdaD is separated from this block. No tdaC homolog is present in any of the *Janthinobacterium* loci. Thus, comparative analysis revealed the absence of one of the genes of the canonical TDA biosynthetic cluster (tdaC) in the operon of *Janthinobacterium* sp. PLB04, while the strain retained the ability to synthesize TDA.

### 2.4. Sequence Similarity and Phylogenetic Analysis of TDA Biosynthetic Proteins

**Figure 4:**
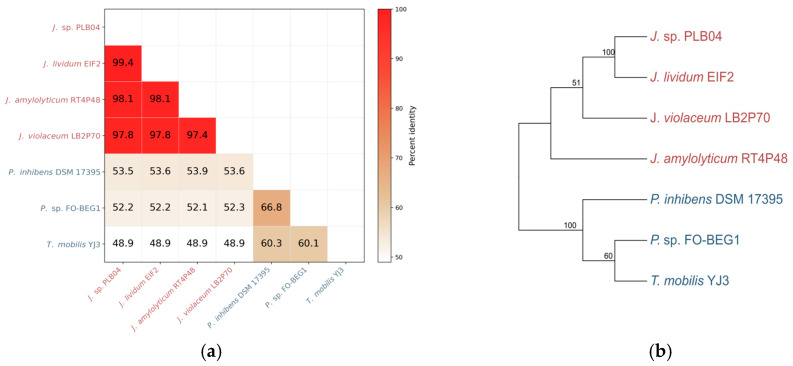
Sequence similarity and phylogenetic relationships of the TDA biosynthetic proteins: (**a**) pairwise amino acid sequence identity (%) based on concatenated TdaA, TdaB, TdaD, TdaE, and TdaF proteins; (**b**) phylogenetic tree constructed from the same concatenated protein sequences using the Maximum Likelihood method (WAG+G model). Bootstrap values (%) are indicated at branch nodes. *Janthinobacterium* and alphaproteobacterial sequences form distinct clusters.

Pairwise sequence identity based on concatenated TdaA, TdaB, TdaD, TdaE, and TdaF proteins showed high similarity among *Janthinobacterium* strains (97.4–99.4%) and lower identity relative to alphaproteobacterial TDA producers (48.9–53.9%) (Figure 4a). Phylogenetic analysis of the same concatenated protein set using the Maximum Likelihood method (WAG+G model) resolved *Janthinobacterium* and alphaproteobacterial sequences into two distinct clusters (Figure 4b). Bootstrap support values confirmed the stability of the observed clustering.

Both sequence identity analysis and phylogenetic reconstruction consistently demonstrated a clear separation between *Janthinobacterium* and alphaproteobacterial TDA-producing lineages.

### 2.5. Experimental Confirmation of TDA Production in Janthinobacterium sp. PLB04

**Figure 5:**
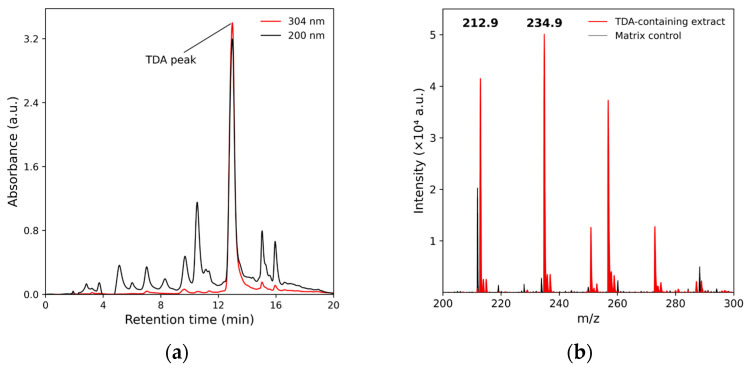
Chromatographic and mass spectrometric confirmation of TDA production by *Janthinobacterium* sp. PLB04: (**a**) HPLC chromatogram of the ethyl acetate extract showing a peak with a retention time of 13 min and an absorption maximum at 304 nm, characteristic of TDA; (**b**) MALDI-TOF mass spectrum of the same extract confirming the presence of tropodithietic acid and its sodium salt.

Cell-free supernatants were analyzed by HPLC to confirm TDA production. Chromatographic analysis of the ethyl acetate extract revealed a single peak with a retention time of 13 min and an absorption maximum at 304 nm (Figure 5a), characteristic of the TDA [12].

Mass spectrometric analysis further confirmed that this peak corresponds to tropodithietic acid, showing signals consistent with TDA and its sodium salt (Figure 5b).

We demonstrated that *Janthinobacterium* sp. PLB04 produces TDA, and extraction from cell-free culture medium sequentially with ethyl acetate, then sodium bicarbonate solution, followed by acidification and re-extraction with ethyl acetate yields an enriched sample suitable for TDA identification by mass spectrometry.

## 3. Discussion

Bacterial tropone natural products, including tropolone, roseobacticides, and TDA, play important ecological roles as antibiotics, signaling molecules, and virulence factors. TDA exhibits antimicrobial activity against a broad range of bacteria and has been associated with disruption of the proton motive force [4].

The biosynthesis of TDA has been shown in marine Alphaproteobacteria of the *Roseobacter* clade and is typically associated with the tdaABCDEF operon [7,10]. Additional genes previously linked to TDA production, such as paaIJK and tdaH, are not physically associated with the main biosynthetic locus and are likely involved in primary metabolism rather than constituting essential components of the biosynthetic system [11].

A key connection between TDA biosynthesis and central metabolism is the phenylacetate (PAA) degradation pathway. In this pathway, the bifunctional enzyme PaaZ plays a central role: its enoyl-CoA hydratase (ECH) domain catalyzes a key ring-opening reaction of phenylacetyl-CoA derivatives, and its NADP^+^-dependent aldehyde dehydrogenase (ALDH) domain subsequently oxidizes the resulting semialdehyde intermediate, ultimately contributing to the conversion of these compounds into central metabolites [13,14]. However, in *P. inhibens*, a modified or functionally impaired variant of this enzyme (PaaZ2) is unable to efficiently perform the ALDH step. As a result, the intermediate 3-oxo-5,6-dehydrosuberoyl-CoA semialdehyde may accumulate. It has been proposed that this intermediate may undergo spontaneous Knoevenagel condensation to form 2-hydroxycyclohepta-1,4,6-triene-1-formyl-CoA, a putative precursor of tropone derivatives [14,15]. If this proposal is correct and the intermediate can undergo spontaneous chemical transformations, then the enzymatic dehydration function attributed to TdaC can be excluded. This uncertainty leaves open the possibility that the role attributed to TdaC may be replaced or complemented by alternative enzymatic reactions.

TdaC has previously proposed to function as a dehydratase involved in the conversion of PAA-derived intermediates into tropone precursors based primarily on bioinformatic annotation rather than direct biochemical evidence [16]. In this context, the proposed substrate of TdaC is likely the same semialdehyde intermediate (or its derivatives) generated during PAA catabolism. However, its precise biochemical role remains unresolved. Importantly, the biochemical function of TdaC remains considerably less well characterized compared to key enzymes such as TdaE and PatB, for which direct mechanistic and enzymatic evidence is available [14,17,18]. This uncertainty leaves open the possibility that the reaction attributed to TdaC may be bypassed, replaced, or complemented by alternative enzymatic or even spontaneous chemical transformations.

In the present study, we identified a TDA biosynthetic gene cluster in the freshwater bacterium *Janthinobacterium* sp. PLB04. The cluster contains homologs of tdaA, tdaB, tdaD, tdaE, and tdaF, but lacks the canonical tdaC gene (Figure 2, Table S1). Despite the absence of this gene, TDA production by this strain was experimentally confirmed by chromatographic (HPLC) and mass spectrometric (MALDI-TOF MS) analyses (Figure 5). Previous genomic studies have reported the presence of TDA-like gene clusters in non-alphaproteobacterial taxa, including representatives of the genus *Janthinobacterium*, where homologs of several core tda genes were identified, but no clear tdaC homolog was detected based on sequence similarity searches [19,20]. However, the functional significance of such non-canonical clusters remained unclear, since experimental studies of TDA production were not conducted. In contrast, this study provides experimental evidence of TDA production in a strain lacking a tdaC homologue. These results confirm previous genomic observations and suggest that TDA biosynthesis in *Janthinobacterium* strain may occur without the canonical tdaC gene, although they do not rule out the possibility of functional replacement of the tdaC by another, unidentified gene.

The gene cluster organization in *Janthinobacterium* differs from that of marine Alphapoteobacteria, including altered gene order and the presence of additional genes such as patB and paaZ located in close proximity to the primary gene cluster. The presence of paaZ-like genes within or near the identified locus further supports the involvement of PAA-derived intermediates and is consistent with current models of TDA biosynthesis that emphasize the integration of secondary metabolism with central aromatic compound degradation pathways [11,15]. Notably, the identified paaZ homolog may contain functionally relevant differences compared to canonical PaaZ proteins, which could indicate a potential functional analogy to the inactive PaaZ2 variant described in *P. inhibens*, although this requires further biochemical validation.

The ability of *Janthinobacterium* sp. PLB04 to produce TDA in the absence of TdaC strongly suggests that the canonical tdaC gene is not strictly required for TDA biosynthesis in this strain, and that formation of the tropone precursor occurs non-enzymatically, in accordance with the scheme described in [14]. Alternatively, the function typically attributed to TdaC may be performed by an as-yet-unidentified enzyme or a functionally unrelated protein encoded outside the canonical cluster. Furthermore, the presence of a highly divergent functional analogue of tdaC elsewhere in the genome cannot be ruled out. Similar functional substitutions have been described in other secondary metabolite biosynthetic pathways and may reflect the broader evolutionary plasticity of these systems [21,22]. Overall, these results suggest that the TDA biosynthetic pathway may be more flexible than previously assumed. The identification of a non-canonical TDA gene cluster in a freshwater Betaproteobacterium expands the known diversity of TDA-producing bacteria and suggests that alternative biosynthetic strategies may be more widespread than currently recognized. In addition, given the known influence of environmental factors such as iron availability on TDA production [23], the occurrence of TDA-like pathways in diverse ecological contexts may be underestimated, particularly in freshwater systems.

The ability of the *Janthinobacterium* sp. PLB04 to produce TDA under freshwater conditions may indicate potential applications; however, further studies are required to evaluate the safety and efficacy of this strain. Therefore, the identification of TDA biosynthesis in bacteria from different taxonomic lineages or ecological niches may significantly expand our understanding of the diversity and evolution of this biosynthetic pathway.

## 4. Materials and Methods

### 4.1. Isolation of the Janthinobacterium sp. PLB04

Samples of diseased *L. baikalensis* sponges were collected from Lake Baikal (Central Siberia), Russia, to obtain cell cultures of the primmorphs, according to the method described earlier [24]. The strain was isolated from the cell cultures of diseased primmorphs. We cultivated bacterial cells on nutrient media including LB broth and R2A agar with 0.05% yeast extract, 0.05% protease peptone, 0.05% tryptone, 0.05% glucose, 0.05% soluble starch, 0.03% sodium pyruvate, 0.03% K_2_HPO_4_, 0.005% MgSO_4_, 1.5 agar, and final pH 7.2 (HiMedia, Mumbai, India). The dishes were inoculated in three repetitions and cultivated for 7 days, at a temperature of 21 °C. Then, colonies of the strain grown on R2A agar were inoculated into 200 mL of liquid LB medium (HiMedia, Mumbai, India) and cultured more than 30 days at 21 °C. Bacterial cells growth was monitored daily. The bacteria cells were stained with a NucBlue Live ReadyProbes reagent (Thermo Fisher Scientific Inc., Waltham, MA, USA). Cell morphology was determined by light microscopy equipped with fluorescence optics (Olympus IX73SC, Olympus Corp., Tokyo, Japan).

### 4.2. Genome Data and Reference Gene Set

The complete genome sequence of *Janthinobacterium* sp. PLB04 was retrieved from the NCBI GenBank database (accession number NZ_CP088968.1). For comparative analysis, genomes were selected based on the following criteria: (i) experimentally confirmed TDA-producing strains, (ii) organisms previously reported or predicted to harbor TDA biosynthetic genes, and (iii) organisms phylogenetically related to the *Janthinobacterium* species in which the presence of the TDA pathway had not been established. Selected genomes included *P. inhibens* DSM 17395, *Pseudovibrio* sp. FO-BEG1, *Tritonibacter mobilis* YJ3, *J. lividum* EIF2, *J. violaceum* LB2P70, and *J. amylolyticum* RT4P48. The analyzed genomes and accession numbers are listed in Table S1.

All genome sequences were used as provided in GenBank and correspond to publicly available annotated assemblies at the time of analysis. Reference protein sequences for canonical TDA biosynthetic genes were obtained from *P. inhibens* DSM 17395. The query set included TdaA, TdaB, TdaC, TdaD, TdaE, and TdaF proteins and is summarized in Table S2.

### 4.3. Identification of TDA-Related Genes and Biosynthetic Regions

Putative biosynthetic gene clusters were initially identified using the antiSMASH web server (version 8.0) [25] with default settings, including KnownClusterBlast and Pfam-based domain detection. For genomes in which no TDA-related clusters were detected under default parameters, additional analyses were performed using relaxed detection settings; however, no TDA-like clusters were identified in these cases.

Because the TDA-related region was predicted with low confidence, targeted homology searches were performed using both BLASTp and tBLASTn algorithms [26]. BLASTp searches were used to identify protein homologs in annotated genomes, whereas tBLASTn searches were applied to genomic sequences to determine precise genomic coordinates and detect potentially unannotated genes.

Reference TdaA–F protein sequences were used as queries to identify homologous genes in the *Janthinobacterium* sp. PLB04 and in comparative genomes. For each query, the best-scoring hits were recorded along with sequence identity, query coverage, bit score, and E-value. The full set of BLAST results is provided in Table S3.

Homologs were considered reliable when supported by sequence identity above 30% and query coverage above 50%, with E-values below 1 × 10^−5^, unless genomic context suggested otherwise. Hits not meeting these criteria, particularly those lacking localization with other TDA-related genes, were treated as false positives and were not interpreted as functional components of the pathway.

Despite extensive homology searches, no convincing tdaC homolog was identified in *Janthinobacterium* sp. PLB04 or in the analyzed, other strains of the *Janthinobacterium* genomes. Additional, low-scoring matches were occasionally detected but did not meet the defined similarity thresholds and were not colocalized with other TDA-related genes. Importantly, comparative analysis of genomic regions containing TDA-related genes did not reveal any unassigned open reading frames or intergenic regions that could plausibly accommodate a divergent tdaC homolog. Instead, these loci were occupied by other annotated genes, supporting the conclusion that tdaC is absent from the *Janthinobacterium* TDA-related loci. These observations argue against the presence of even a highly divergent tdaC homolog within the identified loci.

### 4.4. Comparative Analysis of TDA Gene Organization

The genomic organization of TDA-related genes was analyzed by manual inspection of genomic regions containing identified homologs. Gene order, orientation, and relative distances were assessed based on genomic coordinates obtained from GenBank annotations and tBLASTn mapping. Genes were considered part of the same TDA-related locus when located in close genomic proximity (typically within 10–20 kb) and showing conserved synteny with known TDA gene arrangements. Isolated homologs located outside these regions were not considered components of the biosynthetic system.

Comparative analysis was performed across the *Janthinobacterium* genomes and representative alphaproteobacterial strains. Special attention was given to the arrangement of core genes (tdaA, tdaB, tdaD, tdaE, and tdaF), the absence of tdaC, and the presence of neighboring genes potentially associated with sulfur metabolism and precursor supply, including patB, glutamine amidotransferase, gshB-like genes, and paaZ. In cases of discrepancies between GenBank annotations and tBLASTn results, gene boundaries were interpreted based on sequence similarity and genomic context. Gene cluster diagrams were generated using custom Python (v3.12) scripts based on genomic coordinates.

### 4.5. Sequence Similarity and Phylogenetic Analysis

Amino acid sequences of TdaA, TdaB, TdaD, TdaE, and TdaF were extracted from each analyzed genome and concatenated in a fixed order to represent the conserved core of the TDA biosynthetic system. TdaC was excluded due to the absence of reliable homologs. Multiple sequence alignment was performed using MUSCLE [27] with default parameters. Pairwise identity values were calculated from the aligned concatenated sequences.

Phylogenetic analysis was performed using the Maximum Likelihood method implemented in MEGA11 [28]. The analysis was based on the concatenated amino acid alignment described above, filtered using Gblocks to remove positions with more than 50% gaps. Tree reconstruction was carried out under the WAG+G model (four discrete gamma categories), and branch support was assessed using 1000 bootstrap replicates.

### 4.6. TDA Extraction and Chemical Analysis

The bacteria biomass was separated, and the brown substrate containing a small number of bacteria was centrifuged at 5000× *g* for 20 min. The resulting dark-colored supernatant was stored at 3–5 °C. The extraction of the TDA 100 mL of the substrate was acidified to pH 2 by adding concentrated sulfuric acid and extracted twice with 50 mL of ethyl acetate. The resulting ethyl acetate solution was washed with water (2 × 50 mL). The TDA was extracted from the resulting ethyl acetate solution with two portions of 40 mL and 10 mL of 2% NaHCO_3_ solution. The upper ethyl acetate layer was purple, and the lower soda layer was brown. The soda layer was again acidified by adding 280–300 µL of concentrated sulfuric acid to pH 2 and extracted with two portions of 40 mL and 10 mL of ethyl acetate. The ethyl acetate portions were combined, washed with water, and dried by rotary evaporation. The resulting oily residue was dissolved in 1 mL of acetonitrile and evaporated to dryness several times to remove residual water. The oily residue was dissolved in 500 µL of acetonitrile for chromatographic analysis.

Chromatography was performed on a Milichrom-A02 chromatograph (Ekonova, Novosibirsk, Russia). Column: 2 × 75 mm; Nucleosil 5-C18. Eluent A: H2O/HCOOH (0.1, *v*/*v*) (pH 2.8); Eluent B: MeCN/HCOOH (0.1, *v*/*v*). Gradient: 20% B in 500 µL, then a linear increase in B from 5% to 100% in 2700 µL; re-equilibration to initial conditions (6 min). Flow Rate: 100 µL/min. Column Temperature: 35 °C. UV Detector: 200, 218, 260, 280, 304, 340, 355 nm.

MALDI analysis of crude TDA extract was performed using a matrix-assisted laser desorption/ionization time-of-flight mass spectrometry (MALDI-TOF MS) UltrafleXtreme instrument (Bruker Daltonics, Bremen, Germany). One microliter of the resulting solution in acetonitrile was applied in triplicate to an MTP AnchorChip 384TF S/N 21525MALDI metal plate (Bruker Daltonics, Germany). The dried sample was overlaid with 1 μL of a DHAP matrix solution (10 mg 2,5-Dihydroxyacetophenone dissolved in 50% acetonitrile).

## 5. Conclusions

In this study, TDA production was shown by freshwater bacteria of the strain *Janthinobacterium* sp. PLB04 isolated from the diseased Baikal sponge *L. baikalensis* using cell cultures of the primmorphs. Genome mining revealed the presence of a TDA biosynthetic gene cluster containing homologs of tdaA, tdaB, tdaD, tdaE, and tdaF, while the canonical tdaC gene was absent. Despite the absence of this gene, chromatographic and mass spectrometric analyses confirmed the production of TDA by the strain. Comparative analysis showed that the organization of the identified cluster differs from the canonical TDA operons described in marine bacteria of the *Roseobacter* clade. These findings suggest that TDA biosynthesis in this strain may involve alternative enzymatic mechanisms and that a canonical tdaC gene may not be strictly required for TDA production. The discovery of a TDA-producing strain outside the typical *Roseobacter* lineage expands the known diversity of bacteria capable of producing this compound and provides new insights into the variability and potential flexibility of the TDA biosynthetic pathway. These results demonstrate the applicability of integrated extraction, chromatographic, and mass spectrometric approaches for the identification of small bioactive compounds in newly characterized bacterial strains.

## Data Availability

The genome sequence of *Janthinobacterium* sp. PLB04 analyzed in this study is available in the NCBI GenBank database under accession number NZ_CP088968.1. Other data supporting the findings of this study are available from the corresponding author upon request.

## Supplementary Materials

The following supporting information can be downloaded at: https://www.mdpi.com/article/10.3390/ijms27094052/s1.

## Abbreviations

The following abbreviations are used in this manuscript:

TDA	Tropodithietic acid
PAA	Phenylacetic acid
ECH	Enoyl-CoA hydratase
ALDH	Aldehyde dehydrogenase
HPLC	High-performance liquid chromatography
MALDI-TOF MS	Matrix-assisted laser desorption/ionization time-of-flight mass spectrometry
BLAST	Basic Local Alignment Search Tool
BLASTp	Protein–protein BLAST
tBLASTn	Protein-to-nucleotide translated BLAST
NCBI	National Center for Biotechnology Information
ORF	Open reading frame
PAA pathway	Phenylacetate degradation pathway
